# Histological and histometrical evidences for phenol immunotoxicity in mice

**DOI:** 10.1007/s00580-012-1645-9

**Published:** 2012-11-17

**Authors:** Ali Louei Monfared, Afsaneh Jaafari, Mohammad Taghi Sheibani

**Affiliations:** 1 Department of Basic sciences, Faculty of Para-Veterinary Medicine, University of Ilam, Ilam, Iran; 2 Department of Basic sciences, Faculty of Veterinary Medicine, Ferdowsi University of Mashad, Mashad, Iran; 3 Department of Basic Sciences, Faculty of Veterinary Medicine, University of Tehran, Tehran, Iran; 4 Banjonjab St-Pajhoohesh St-Ilam University, Ilam, Iran 69315-516

**Keywords:** Histology, Phenol, Immunotoxicity, Mice

## Abstract

Phenol is a common industrial and ubiquitous environmental chemical which is used to synthesize resins and plastics. Due to its anesthetic and disinfectant properties, phenol is also widely used in pharmaceutical products. Since there were no adequate data about phenol immunotoxicity, the purpose of the present study is to investigate its toxic effects on the histological structures of the lymphoid organs in the mice. A total of 80 mice were randomly distributed into one control group and three experimental groups. The control group received only distilled water, whereas experimental groups were orally administered phenol at the concentrations of 80, 180, and 320 mg/kg/day, respectively. After 28 consecutive days, tissue samples were taken and histological changes of the spleens, thymuses, adrenal glands, and lymph nodes were examined using optical microscopy. The results showed that in the phenol treated animals; splenic megakaryocyte counts increased, the diameter of the splenic follicles decreased, the thymocyte population in both cortex and medulla reduced, the thickness of the reticular layers of adrenal gland increased and lymphatic cells populations in the lymph node were reduced, significantly (*P* < 0.01). Also, remarkable histological changes were noted in the various lymphatic organs of the treated mice. Overall, present findings give some histological evidences that selected qualitative and quantitative parameters of the lymphatic organs were significantly altered by phenol administration. In conclusion, the significant decreases of the immune cell populations together with histological alterations in the immunocompetent organs of the mice exposed to phenol indicate the immunosuppressive and immunotoxic properties of this chemical material.

## Introduction

Phenol (C_6_H_5_OH), a monohydroxy derivative of benzene, is used to synthesize resins and plastics (Finkelstein et al. 2007). Also, large amounts of phenol produced in industry and from other natural sources cause this chemical to be an important environmental and occupational hazard (Bruce et al. 1987). Due to its anesthetic and disinfectant properties, phenol is also widely used in pharmaceutical products such as ointments, ear and nose drops, sprays, and antiseptic lotions (Finkelstein et al. 2007).

The detrimental health effects attributed to phenol toxicity in the humans or animals include renal toxicity (Tootian et al. 2012), hematotoxicity (Louei Monfared and Salati 2012; Baj et al. 1994), immunotoxicity (Hsieh et al. 1988; Baj-Zeman et al. 1990), and neurological disorders (Windus-Podehl et al. 1983).

Tootian et al. (2012) investigated the nephrotoxic properties of the phenol in the mice. The cytotoxicity of phenol was attributed to phenoxyl-type radical production from phenol and their ability to impair epithelial cell membrane integrity (Tootian et al. 2012).

Baj et al. (1994) reported that in the workers who had been exposed for 6 months to vapors of phenol, formaldehyde, and organic chlorohydrocarbons; the number of T-lymphocytes and NK cell cytotoxicity were significantly decreased and lymphocyte proliferation was diminished. Also, subjects with the highest levels of phenol in the urine had decreased T-helper lymphocyte numbers and increased numbers of eosinophils and monocytes. The authors concluded that the functions of both the immune and hematopoietic systems could be affected by chronic exposure to these toxic substances (Baj et al. 1994).

Hsieh et al. (1988) reported immunotoxic effects of oral benzene (a parent chemical of phenol) exposure in the mice. They demonstrated that the immunotoxicity is the result of benzene metabolites including phenol.

Although the understanding of the toxic actions of phenol on the lymphoid organs is very important for occupational and public health, however, to the best of our knowledge, there is not a comprehensive study on histological and histometrical changes of the lymphoid organs after phenol exposure. So, the purpose of the present study was to investigate the effects of this chemical material on histological structures of lymphoid organs in the mice.

## Materials and methods

### Chemical

Phenol (C6H5OH) was obtained from Biochem Chemical Co. (Tehran, Iran) and dissolved in distillated water. Deionized distilled water was used as the phenol career. Solutions of this chemical material at concentrations of 80, 180, and 320 mg/kg were prepared to provide the appropriate doses for experiments.

### Animal and experimental design

To do the experiments, a total of 80 male Balb/C mice, 9–10 weeks old, were purchased from Razi Institute (Karaj, Iran). The animals were maintained in a controlled environment at a temperature of 23 ± 1 °C, natural 12:12 h light–dark cycle, and had ad libitum access to drinking water and food. Animals were allowed to be acclimatized to the laboratory environment for at least 7 days before commencement of testing. The mice were randomly allotted into four equal groups (*n* = 20); in which mice were exposed to different doses of phenol. The experiment was carried out for 28 consecutive days and animals were randomly divided into one control group and three experimental groups, each comprising of 20 mice. The control group received only distilled water, whereas experimental groups were orally administered phenol at the concentrations of 80, 180, and 320 mg/kg/day, respectively. The concentrations were determined on the basis of a primary study. Also, the concentrations and stabilities of the chemical were confirmed. All experimental procedures were carried out in accordance with institutional guidelines for animal care and use at the University of Ilam.

### Histological and histometrical assessment

At the end of the administration period; the animals were anesthetized with chloroform vapor, quickly brought out of the jar, and sacrificed. For tissue assessment; the specimens from spleens, thymuses, adrenal glands, and sub-iliac lymph nodes were immersion imprisoned overnight in 10 % neutral buffered formalin to be fixed. Then the specimens were mounted to allow 5-μm sections. Sections were stained via hematoxylin and eosin method and photographed directly using a stereo microscope in 400 high power fields with Microsoft system.

For exact description of the structural changes in the lymphatic tissues, a histometrical analyze was performed. For this purpose, splenic megakaryocytes in unit area of (1.44 × 10^4^ μm^2^ tissue area) were determined by counting in 10 randomly selected areas in subcapsular white pulp regions (Salbacak et al. 2001) using Image Tool® 3.0 software (UTHSCSA, San Antonio, TX, USA). Also, in each animal from all of the groups 10 tissue sections (7 μm) were taken at 21 μm intervals, and splenic capsule thickness, and also splenic follicular diameter were recorded. Furthermore, thymic capsule thickness, thymic cortex diameter, as well as thymic medulla diameters were recorded. In addition, the thickness of the glomerular, fascicular, reticular, and medullary layers of adrenal glands has been determined. Finally, the thickness of the lymph node’s capsule and the diameter of the lymph node’s follicles were determined according to Salbacak et al. (2001).

### Data analysis and statistics

All results were expressed as standard error of the mean. The analysis of variance was used to test the overall significance of differences among the means. Tukey–Kramer’s multiple comparison test was applied for post hoc comparison. Computations were performed using Statistical Package for Social Scientist (SPSS 11.5). A probability level of less than 5 % (*P* < 0.05) was considered as significant.

## Results

### Spleen

**Fig. 1:**
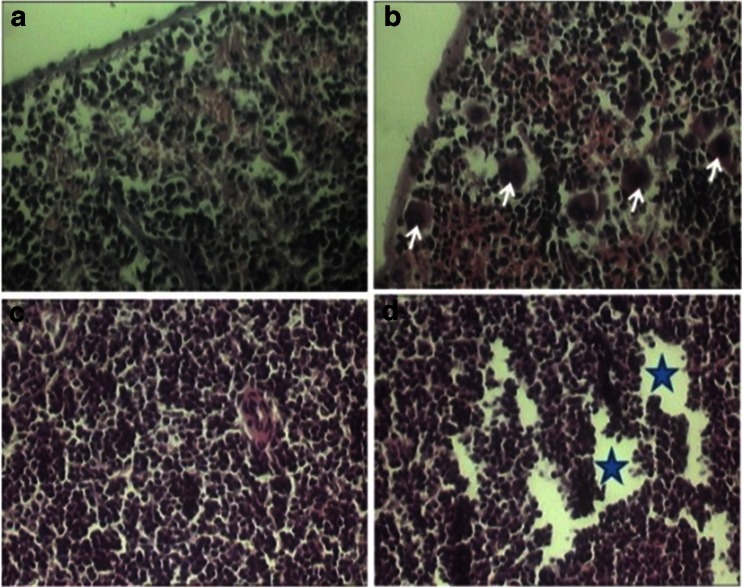
Spleen transverse sections of the control (**a**, **c**) and 320 mg/kg phenol treated (**b**, **d**) animals. The **b** section shows abundant megakaryocytes (*arrows*) infiltration in the whole parenchyma of the spleen in the phenol-treated mice. The **d** section shows severe depletion (star area) of the different lymphoid cells in the whole parenchyma of the spleen in the phenol-treated mice (hematoxylin and eosin stain; **a**–**d** ×400)

In the phenol-treated animals, spleen was severely affected; so that the splenic megakaryocyte counts have significantly increased in the treatment group (Table 1) in comparison with control animals (*P* < 0.01) (Table 1). In these animals, both histological (Fig. 1d) and morphometric (Table 1) results have demonstrated severe splenic lymphocyte depletion. Although, the thickness of the spleen’s capsule was not affected by phenol administration (Table 1) but the diameter of the splenic follicles showed a significant decrease in the phenol-treated animals compared to those of the controls (*P* < 0.01; Table 1). It was not found any outstanding differences in the histological results between three experimental groups.

**Table 1:** Summarized histometric changes in spleens, thymuses, adrenal glands, and sub-iliac lymph nodes of the mice exposed to different concentrations of phenol

	Spleen			Adrenal gland				Thymus			Lymph node	
Parameters/Groups	SCT (μm)	GCD (μm)	MC/UA	GLT (μm)	FLT (μm)	RLT (μm)	MT (μm)	TCT (μm)	TMD (μm)	TCD (μm)	LCT (μm)	LFD (μm)
Control	4.2 ± 1.3	67.6 ± 8.7	2.3 ± 0.60	98 ± 1.3	98 ± 3.1	82.3 ± 2.9	51.1 ± 5.7	18.4 ± 6.3	51.4 ± 1.1	78.8 ± 9.8	14.2 ± 1.3	74.6 ± 9.0
80	7.0 ± 3.7	43.9 ± 1.7	5.0 ± 1	96 ± 2.4	102 ± 6	71.6 ± 9.1	50.1 ± 5.6	18.5 ± 2.7	45.7 ± 1.6	41.7 ± 1.2	13.1 ± 1.6	40.2 ± 6.2
180	4.2 ± 1.3	37.7 ± 5.4	6.0 ± 1.7	81 ± 1.3	96.8 ± 8.7	81.1 ± 8.7	54.1 ± 0.80	18.6 ± 3.9	45.4 ± 8.6	40.1 ± 6.0	12.8 ± 2.1	34.8 ± 2.9
320	8.1 ± 3.7	39.0 ± 5.6	8.7 ± 0.60	89 ± 3.2	108 ± 4.9	102 ± 7.5	51.7 ± 4.8	17.1 ± 5.8	44.6 ± 1.3	51.2 ± 1.9	13.4 ± 1.5	35.7 ± 1.6
Significance	–	**	**	–	–	**	–	–	–	**	–	**

*SCT* splenic capsule-thickness, *GCD* the diameter of germinal center of the lymphoid follicles, *MC/UA* megakaryocyte count/unit (1.44 × 104 μm2) tissue area, *GLT* glomerular layer thickness of adrenal, *FLT* fascicular layer thickness of adrenal, *RLT* reticular layer thickness of adrenal, *MT* medullary layer thickness of adrenal, *TCT* thymic capsule thickness, *TMD* thymic medulla diameter, *TCD* thymic cortex diameter, *LCT* lymph node capsule thickness, *LFD* lymph node follicular diameter

*P < 0.05

**P < 0.01

### Thymus

**Fig. 2:**
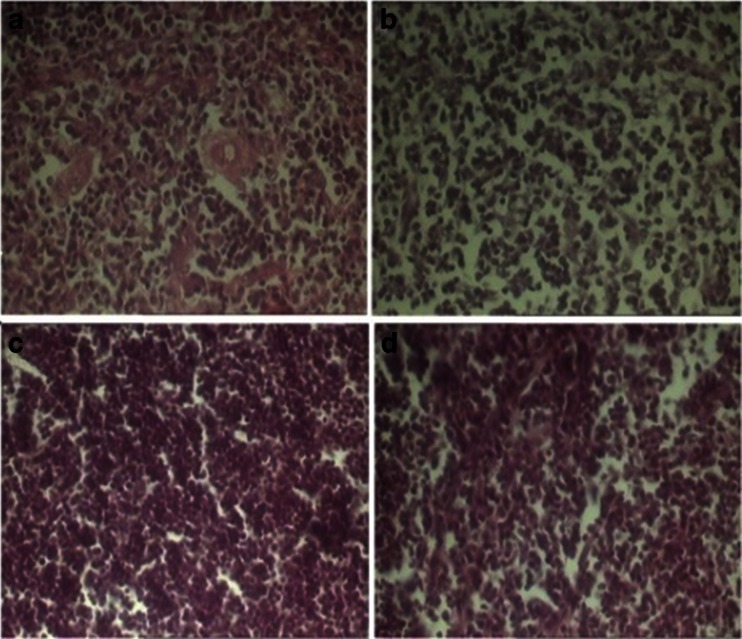
The transverse sections of the medulla of thymus in the control (**a**), the medulla of thymus in the 180 mg/kg phenol-treated (**b**) animals, the cortex of thymus in the control (**c**), and the cortex of thymus in the phenol-treated (**d**) animals. The figure shows that in the mice treated with phenol; severe depletion of the lymphoid cells from both cortex and medulla occurred (hematoxylin and eosin stain; **a**–**d** ×400)

Thymus tissue of the control group had normal cellular population in its cortical and medullar compartments (Table 1) while phenol treated mice demonstrated a reduction of the thymocyte population in the both cortex and medulla (Fig. 2c, d). In the mice administered with phenol, the diameter of the thymic cortex were significantly lower than those of the controls (*P* < 0.01; Table 1) while decreasing of the diameter of thymic medulla in those animals was not significant statistically. Also, the thickness of the thymus’s capsule was not affected by phenol administration (Table 1).

### Adrenal glands

**Fig. 3:**
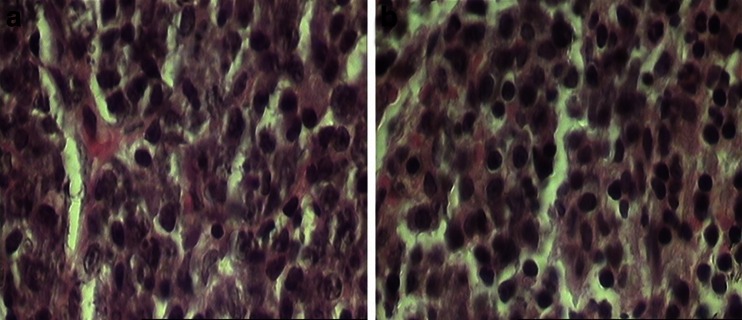
The transverse sections of the reticular layer of adrenal cortex in the control (**a**) and 320 mg/kg phenol-treated (**b**) animals. The **b** section shows significant increase of the lymphoid cells in the reticular layer of the adrenal glands in the phenol-treated mice (hematoxylin and eosin stain; **a**, **b** ×400)

In the control group, the adrenal glands have displayed normal histology with a large cortical and medullar compartments. But adrenal glands structures in experimental animals have been affected and lymphoid cells in its cortex and especially in reticular layer show significant increasing (*P* < 0.01; Fig. 3b, Table 1). Though, the medullar thickness of the adrenal glands was not changed by phenol treatment.

### Lymph node

**Fig. 4:**
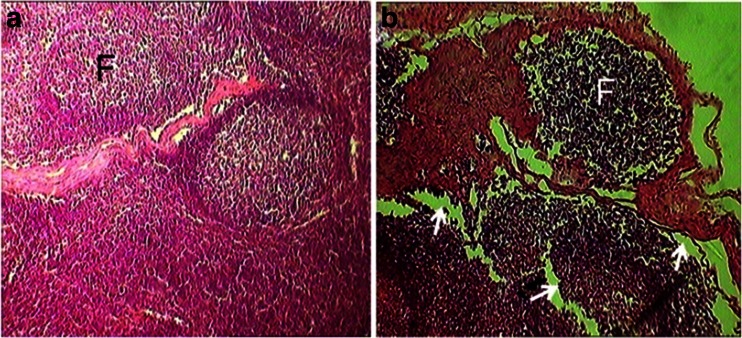
The transverse sections of the sub-iliac lymph node in the control (**a**) and 180 mg/kg phenol-treated (**b**) animals. The b section shows significant decrease in the size of the follicles (*F*) as well as empty spaces (*arrows*) around the follicles in the phenol treated mice (hematoxylin and eosin stain; **a**, **b** ×400)

Sub-iliac lymph nodes of the control mice had typical histological integrity which was include large lymphoid follicles in the cortical area as well as massive lymphatic sinuses in the medullary area. In the treated animals, the tissue integrity of the lymph node was changed and lymphatic cells populations were reduced (*P* < 0.05; Table 1). Furthermore, in the lymph nodes belonging to the experimental animals, empty spaces were seen around the follicles (Fig. 4b). The diameter of the lymph node’s follicles of phenol given animals was also lower than those of the controls (*P* < 0.01; Table 1). In addition, the thickness of the lymph node’s capsule was not affected by phenol treatment (Table 1).

## Discussion

Lymphoid tissues recently have received considerable attention as a target organ for the achieving the chemical materials toxicity (Hsieh et al. 1992). Present findings give some histological evidences that selected qualitative and quantitative parameters of the lymphatic organs were significantly altered by phenol administration.

In the present study, the cellularity of spleen was affected by the phenol administration. In other words, morphometric results have indicated that phenol caused profound suppression in the splenocytes population in the treated mice. Splenic immunosuppression may be attributing to the decreased different lymphatic cells numbers in the spleen as well as other immune organ. In line with these results, similarly, previous studies have demonstrated the immunological alterations on the splenic-forming cells and corresponding circulating antibody levels (Hsieh et al. 1992) in the CD-1 mice. In contrast, no spleen toxicity was observed in the study of Ryan et al. (2001) on the Sprague–Dawley rats. This dissimilarity may be associated with possible interspecies and inter strains differences between rats and mice for this endpoint. In addition, Hsieh et al. (1992) demonstrated that treatment with 19.5 and 95.2 mg phenol/L in the drinking water inhibits the proliferative ability of splenic lymphocytes. Also, Berman et al. (1995) reported that an oral dose 224 mg/kg phenol caused spleen atrophy. The above mentioned findings are in accordance with our results.

In this study, phenol administration caused significant declines (*P* < 0.01) in the cellular population of the thymic cortex. In accordance with this result, Berman et al. (1995) reported that an oral dose 224 mg/kg phenol caused thymus atrophy or necrosis. Similarly, Moszcynski and Lisiewicr (1983) revealed a decrease in the number of T lymphocytes among workers exposed to benzene (a parent chemical of phenol) and several other solvents.

In the present study, overall thickness of the adrenal cortex has increased in the phenol treated animals, although statistical analysis have showed that adrenal reticular zone thickened significantly compared to those of control. Also, in the phenol given mice; the tissue integrity of the lymph node was changed, the diameter of the lymph node’s follicles was reduced and empty spaces were formed around the follicles. This finding means adrenal hypertrophy as well as lymph node atrophy after phenol administration. Adrenal hypertrophy after phenol exposing may be due to hypothalamic–pituitary–adrenal axis activation resulting in high serum level of adrenal corticosteroids (Freier and Fuchs 1994). The atrophy of the lymph node is attributable to the decreased different lymphatic cells numbers in this organ resulting in suppress of the lymphocyte growth after phenol treatment. In this regards, Zeman et al. (1990) has been shown a decrease in the CD3 lymphocytes production together with decreased CD4/CD8 ratio and abnormal values of lymphocyte subpopulations in the workers whom exposed to phenol (Zeman et al. 1990).

In accordance with the results above, it has been demonstrated that phenol is main primary metabolite of benzene in the biological systems (Sawahata and Neal 1983) and benzene cause immunotoxic effects include lymphopenia and leucopenia in the human and animal studies (Avogbe et al. 2011; Wierda et al. 1981). Also, 1.4-dihydroxyphenol induced damages of chromosomes in human lymphocytes, which may led to leukemia progression (Zhang et al. 1998).

Although the mechanism(s) of phenol immunotoxicity are still unclear, but its ability to suppress the lymphocyte growth had been shown correlates with its oxidation capacity and with its concentration in the lymphoid organs and bone marrow (Pfeifer and Irons 1983). In addition, it has been suggested that phenol undergo hydroxylation reaction in the liver, alternatively with the consecutive production of catechol; which is converted to *o*-benzoquinoue. Subsequently, *o*-benzoquinoue derived from catechol is generally considered to be the toxic metabolites (Snyder and Hedli 1996). This phenomenon is other assumable mechanism of action for phenol toxicity.

Beside liver’s enzymes metabolism of phenol, the bone marrow contains several peroxidases and the most prevalent is myeloperoxidase (Subrahmanyam et al. 1991). On the other hand, it has been shown that phenol is transported to the bone marrow, where myeloproxidase is responsible for converting this chemical material to several biologically toxic compounds (Tsuruta et al. 1999). So, the myeloproxidase’s ability to metabolize of phenol to toxic quinines in the bone marrow is other possible mechanism to phenol immunotoxicity.

In conclusion, the significant decreases of the immune cell populations in the immunocompetent organs of the mice exposed to phenol indicate the immunosuppressive and immunotoxic properties of this chemical material. Further studies will be needed to explore exact causative mechanism(s) and factors for phenol-induced immunological toxicity and especially in the search for suitable health surveillance methods.
